# Sentiment Analysis of Students’ Feedback in MOOCs: A Systematic Literature Review

**DOI:** 10.3389/frai.2021.728708

**Published:** 2021-09-09

**Authors:** Fisnik Dalipi, Katerina Zdravkova, Fredrik Ahlgren

**Affiliations:** ^1^ Faculty of Technology, Linnaeus University, Växjö, Sweden; ^2^ Faculty of Computer Science and Engineering, Ss. Cyril and Methodius University, Skopje, North Macedonia

**Keywords:** massive open online courses, MOOCs, sentiment analysis, systematic review, student feedback, learning analytics, opinion mining

## Abstract

In recent years, sentiment analysis (SA) has gained popularity among researchers in various domains, including the education domain. Particularly, sentiment analysis can be applied to review the course comments in massive open online courses (MOOCs), which could enable instructors to easily evaluate their courses. This article is a systematic literature review on the use of sentiment analysis for evaluating students’ feedback in MOOCs, exploring works published between January 1, 2015, and March 4, 2021. To the best of our knowledge, this systematic review is the first of its kind. We have applied a stepwise PRISMA framework to guide our search process, by searching for studies in six electronic research databases (ACM, IEEE, ScienceDirect, Springer, Scopus, and Web of Science). Our review identified 40 relevant articles out of 440 that were initially found at the first stage. From the reviewed literature, we found that the research has revolved around six areas: MOOC content evaluation, feedback contradiction detection, SA effectiveness, SA through social network posts, understanding course performance and dropouts, and MOOC design model evaluation. In the end, some recommendations are provided and areas for future research directions are identified.

## Introduction

Recent innovations in digital learning have provided great opportunities to shift learning pedagogies away from conventional lecture methods toward more creative and effective teaching methods. These methods involve learners in collaborative learning and offer open access to course content to a large scale of learners. One such learning method that has received much attention is the Massive Open Online Courses (MOOCs), whose slogan is: “Education for anyone, anywhere, and any time” (Zemsky, 2014). MOOCs are online courses that offer free access via the Web to a huge number of learners around the world. They introduce interactive user forums that support and encourage collaborative learning and active participation of students (Rabbany et al., 2014). Moreover, their spread and popularity are enabling learners to satisfy the learning expectations and needs in an open, engaging and distributed manner (Littlejohn et al., 2016; Dalipi et al., 2017). Students’ feedback represents an indispensable source of information that can be used by teachers or educational instructors in order to enhance learning procedures and training activities. The popularity and importance of student’s feedback have increased especially in the COVID-19 pandemic times when most educational institutions have transcended traditional face-to-face learning to online format. However, due to the nature of the language used by students and the large volume of information expressing their points of view and emotions about different aspects in MOOCs forums, dealing with and processing the students’ opinions is a complex task. One way to overcome these challenges is by leveraging the advantages of sentiment analysis and opinion mining techniques.

Sentiment analysis, which is the process of finding sentiment words and phrases that exhibiting emotions, has attracted a lot of research attention recently, especially in the education domain in general and in MOOCs in particular (Lundqvist et al., 2020; Onan, 2021). SA systems use natural language processing (NLP) and machine learning (ML) techniques to discover, retrieve, and distill information and opinions from vast textual information (Cambria et al., 2013).

Sentiments can provide a valuable source of information not only for analyzing a student’s behavior towards a course topic, but also for enhancing policies and higher education institutions for their improvement (Kastrati et al., 2021). In this perspective, the past couple of years there has been a trend with increased publications where different sentiment analysis techniques, including NLP, and deep learning (DL), are successfully used for this purpose (Estrada et al., 2020; Zhou and Ye, 2020).

The main goal of this paper is to critically evaluate the body of knowledge related to sentiment analysis of students’ feedback in MOOCs, by answering research questions through a stepwise framework for conducting systematic reviews. By exploring the current state of knowledge in the field, we also demonstrated that the knowledge body of educational technology research lacks a comprehensive and systematic review that covers studies about MOOCs learners’ feedback sentiment analysis. Therefore, our study will try to fill these gaps by analyzing and synthesizing research findings to describe state of the art and provide some valuable guidelines for new research and development efforts in the field.

Furthermore, the findings derived from this review can serve as a basis and a guide for future research and teaching practice as MOOC based teaching is becoming one of the approaches that is widely implemented in traditional curriculum and educational practices of many higher education institutions.

The rest of the paper is organized as follows: *Methodology* describes the search strategy and methodology adopted in conducting the study. *Results and Analysis* presents the systematic review study results. Themes identified from the investigated papers are described in *Discussion*. *Discussion* also outlines recommendations and future research directions for the development of effective sentiment analysis systems. Lastly, final conclusions are drawn in the *Conclusion* section.

## Methodology

For this systematic literature review (SLR) study, the PRISMA guidelines provided in (Liberati et al., 2009) were applied. SLR represents a thorough and comprehensive research method for conducting a literature review in a systematic manner by strictly following well-defined steps. This method is guided by specific research questions; and by being systematic and explicit, it reduces biases in the review process. It also includes applying a structured and stepwise approach and designing a research protocol (Petticrew and Roberts, 2006; Staples and Niazi, 2007; Liberati et al., 2009; Onwuegbuzie et al., 2012). As also reported by Fink (2019), a systematic literature review is an organized, comprehensive, and reproducible method. Using these definitions, the main purpose of this study was to:

• report on previous research works on sentiment analysis applications in MOOC setting, and
• provide an exhaustive analysis that could serve as a platform for future opportunities and paths for research and implementation in the field.

Having these purposes in mind the paper will identify and report the investigated entities/aspects, the most frequently used bibliographical sources, the research trends and patterns, scenarios, architectures, techniques and the tools used for performing sentiment analysis in MOOC.

The following research questions guide this systematic literature review:

• **RQ1**. What are the various techniques, tools, and architectures used to conduct sentiment analysis in MOOCs discussion forums?
• **RQ2**. In what scenarios and for what purpose is the sentiment analysis performed in the selected papers?

### Search Strategy and Data Collection

**FIGURE 1:**
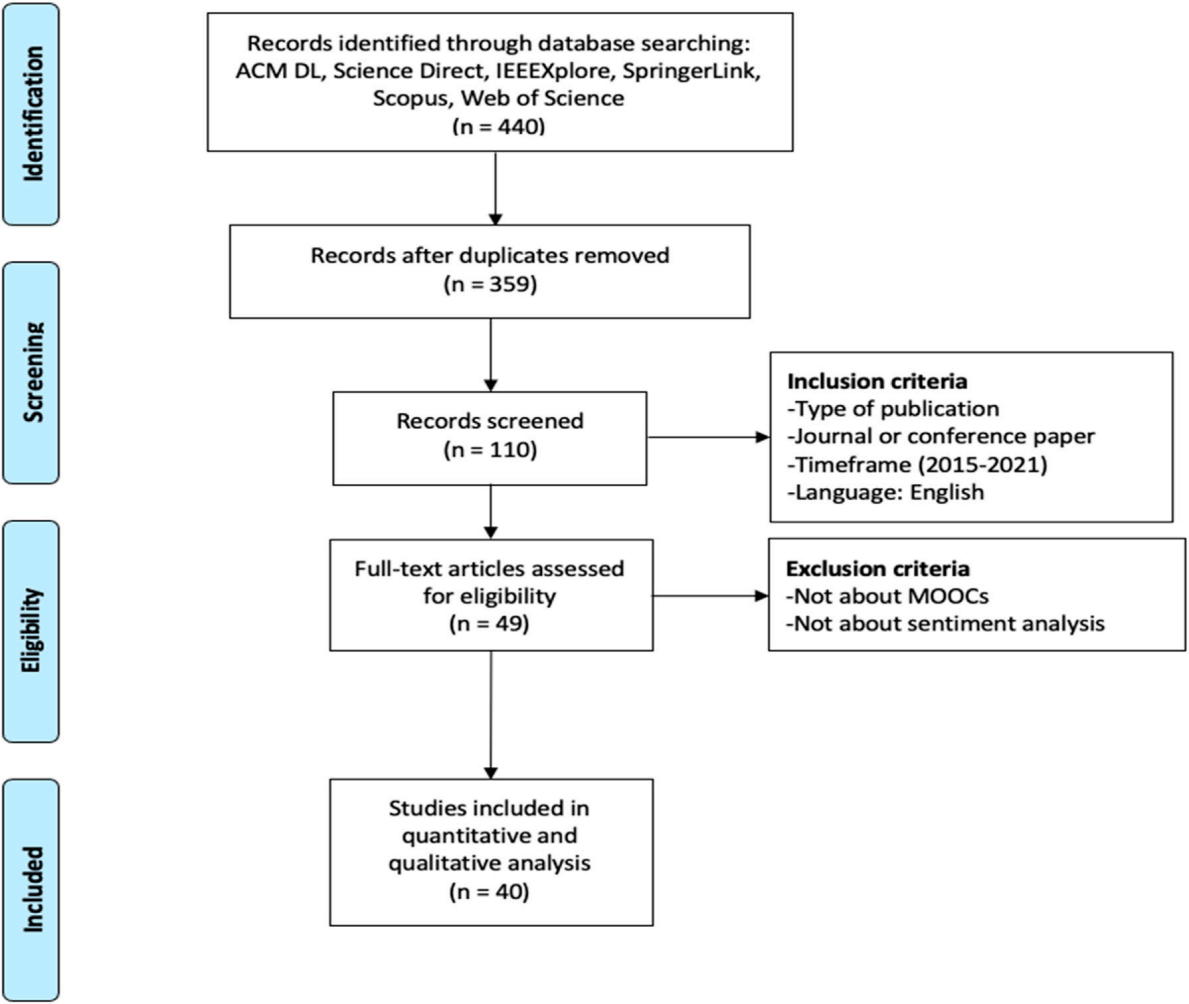
Implemented PRISMA search methodology.

The online JabRef ® software facilitated the article search and selection following the PRISMA approach. To ensure that all relevant studies were collected and reviewed, search strategy involved a stepwise approach that consists of four stages. The overall process of search strategy is shown in Figure 1.

The first stage entails the development of a research protocol by determining the research questions, defining the search keywords and identifying the bibliographic databases for performing the search. For the search purposes, following online research databases and engines were systematically examined: ACM DL, IEEE Xplore, ScienceDirect, Scopus, SpringerLink, and Web of Science. In total, the first stage yielded 440 articles, and after all the duplicates were removed, it produced a reduced list of 359 articles to be processed and included for the upcoming stage of screening.

The keywords used in this study were driven by the PICO framework, and are shown in Table 1. PICO (Population, Intervention, Comparison, and Outcomes) is aimed at helping researchers to design a comprehensive set of search keywords for quantitative research in terms of: Population, Intervention, Comparison, Outcome, and Context (Schardt et al., 2007). As suggested by (Gianni and Divitini, 2015), aiming to avoid missing possible relevant articles, a Context section to the PICO schema was added. Table 2 presents the final search keywords associated with PICO(C) used in the study.

**TABLE 1:** PICO(C) driven keywords framing.

Population	Students (learners)
Intervention (Investigation)	Sentiment analysis or opinion mining
Comparison	—
Outcome (What do we measure or evaluate?)	students’ feedback, opinion mining, sentiment analysis, teacher assessment, user’ feedback, feedback assessment
Context	MOOC

**TABLE 2:** Search string (Query).

Context	“MOOC”
AND	
Intervention	(“sentiment analysis” **OR** “opinion mining”)
AND	
Outcome	(“students’ feedback” **OR** “teacher assessment” **OR** “user feedback” **OR** “feedback assessment” **OR** “students’ reviews” **OR** “learners’ reviews” **OR** “learners’ feedback” **OR** “student ratings” OR “teacher evaluation”)

First, for all the sections of PICO(C) in Table 1 the adequate keywords were identified, followed by the self-constructed search string by applying binary operators, as shown in Table 2. To ensure that any possible relevant article will not be omitted in the study, a context section was also added as a separate feature.

Screening refers to stage 2 of the search strategy process and involves the application of inclusion criteria. At this stage, the relevant studies were selected based on the following criteria: 1) type of publication needs to be a peer-reviewed journal or conference paper, 2) papers should have been published between 2015 and 2021, and 3) papers should be in English. After applying the mentioned criteria in the search process, out of 359 papers, a total number of 110 records were accepted as relevant studies for further exploration. The authors agreed to encode the data using three different colors: 1) green—papers that passed the screening threshold, 2) red—papers that did not pass the screening threshold, and 3) yellow—papers that the authors were unsure which category to classify them as (green or red). For such papers, a comprehensive discussion between the authors took place, and once a consensus was reached, those papers were classified into either the green or red category. 

In Stage 3, which in Figure 1 corresponds to eligibility, the studies that are explicitly not: 1) within the context of MOOC, 2) considering sentiment analysis were eliminated. At this stage, all the titles, abstracts, and keywords were examined to determine the relevant records for the next stage. After these criteria, only 49 papers were considered eligible for future investigation in the last stage of analysis.

Moreover, after carefully reading and observing the eligible papers, it was found that three out of 49 papers were lacking full text, and another 6 papers were either review papers or were only employing tools, without providing rich information on the algorithmic applications for sentiment analysis. Therefore, those papers were also excluded, which decreased the number of eligible papers to 40.

### Limitations

When assessing this systematic literature review, there are several factors that need to be considered, since they can potentially limit the validity of the findings: These factors include:

• Only papers written in English were selected in the study. While searching the research databases, we found related articles in other languages, such as Chinese and Spanish. Those articles are not included.
• The study includes papers collected from the six digital research databases shown in Figure 1. Thus, we might have potentially missed papers having been indexed in other digital libraries.
• For this study, only peer reviewed journal articles, conferences and book sections are selected. Scientific studies that are not-peer reviewed are not included.
• Only works published between January 1, 2015, and March 4, 2021, are selected in this study. We highlight that there may have been conference papers presented before March 4, 2021, that were not published by the cut-off date for this study and that they were not included in our literature review.

## Results and Analysis

**FIGURE 2:**
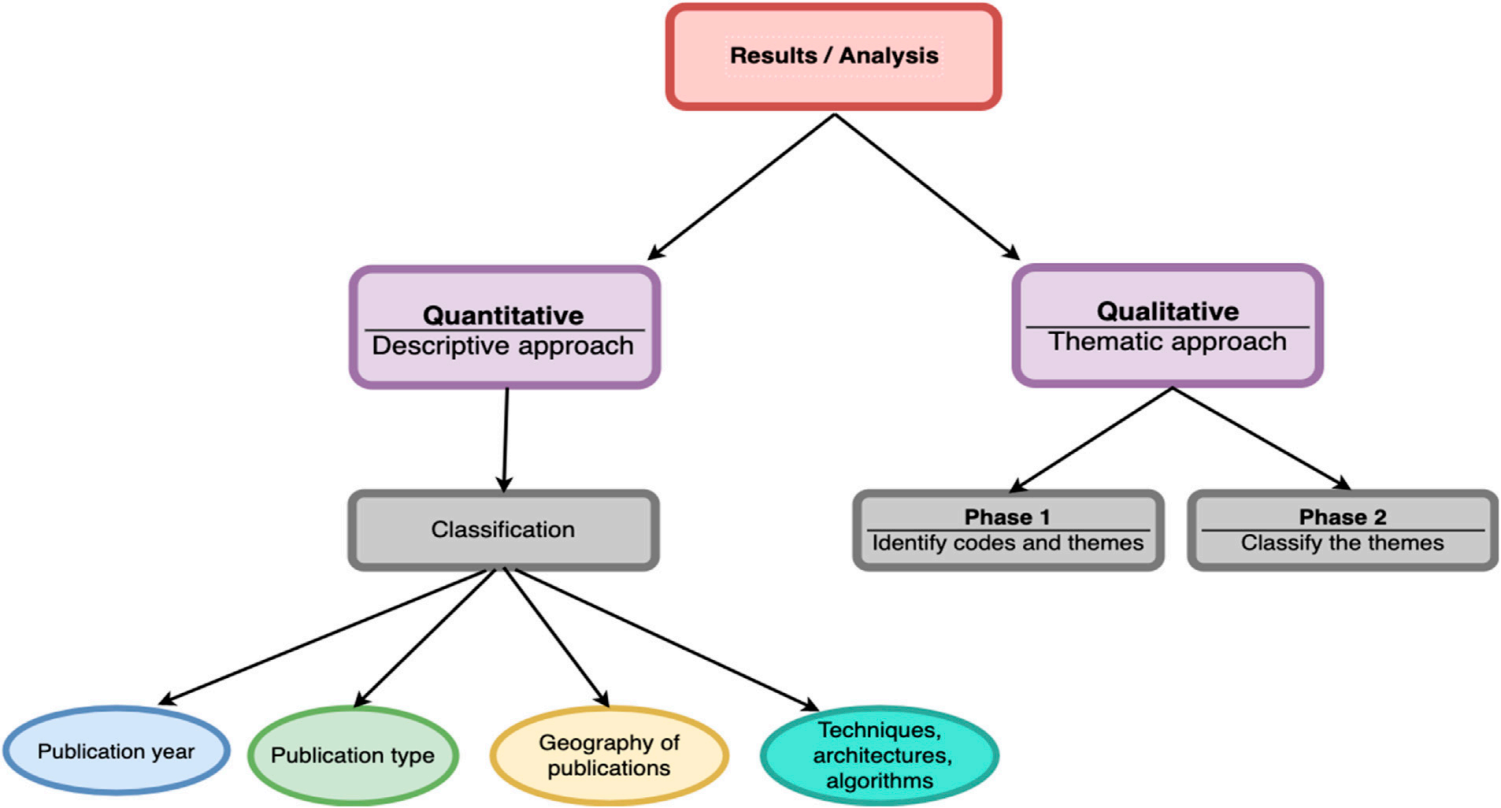
Analysis process of the relevant contributions.

After determining the core set of eligible papers, both quantitative and qualitative analysis on the data were performed. In the quantitative approach, data categorization of the findings was performed, based on the publication year, venue, publication type, geographic region of the authors and also data based on techniques, architectures, algorithms and tools. On the other hand, for qualitative analysis, an open coding content analysis method as described in (Braun and Clarke, 2006) was used. This technique comprises two phases: first, reading all papers to extract themes, and second, classifying the identified themes. The Figure 2 below showcases the process of analysis.

### Quantitative Analysis

**FIGURE 3:**
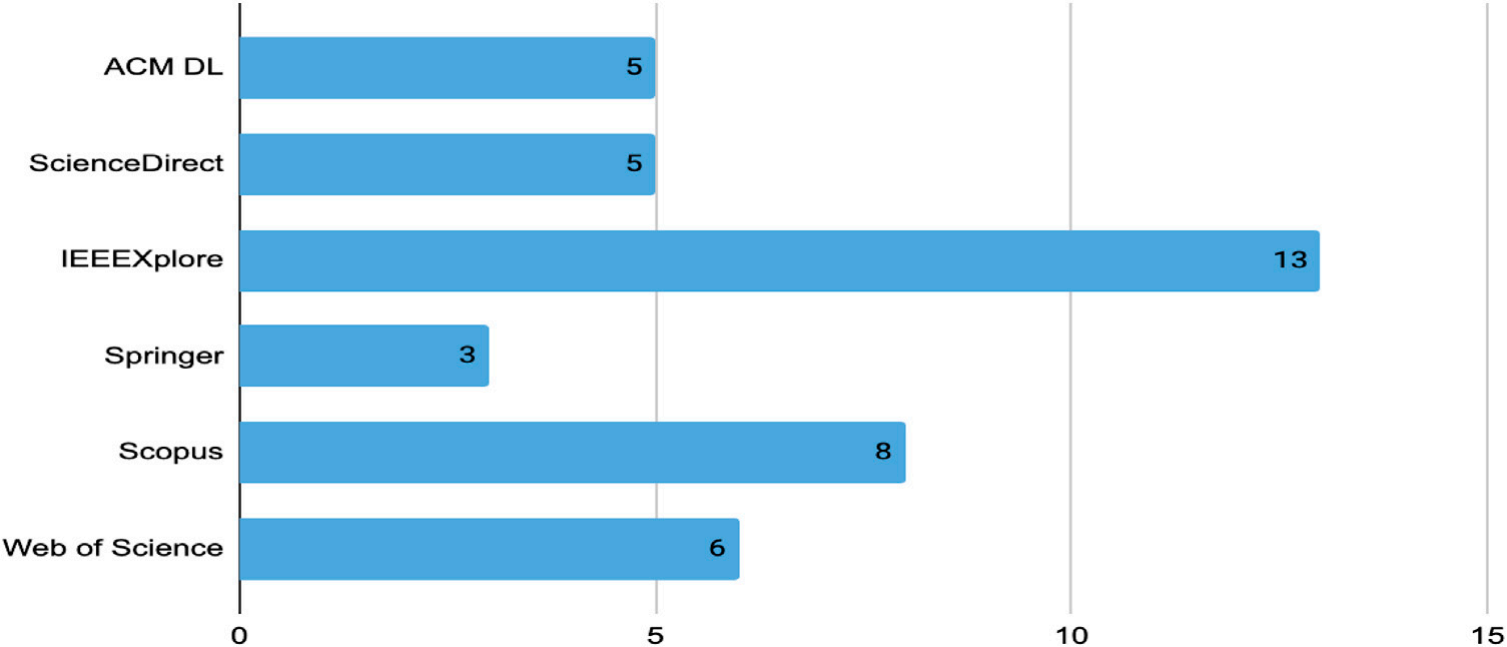
Distribution of studies in academic databases.

**FIGURE 4:**
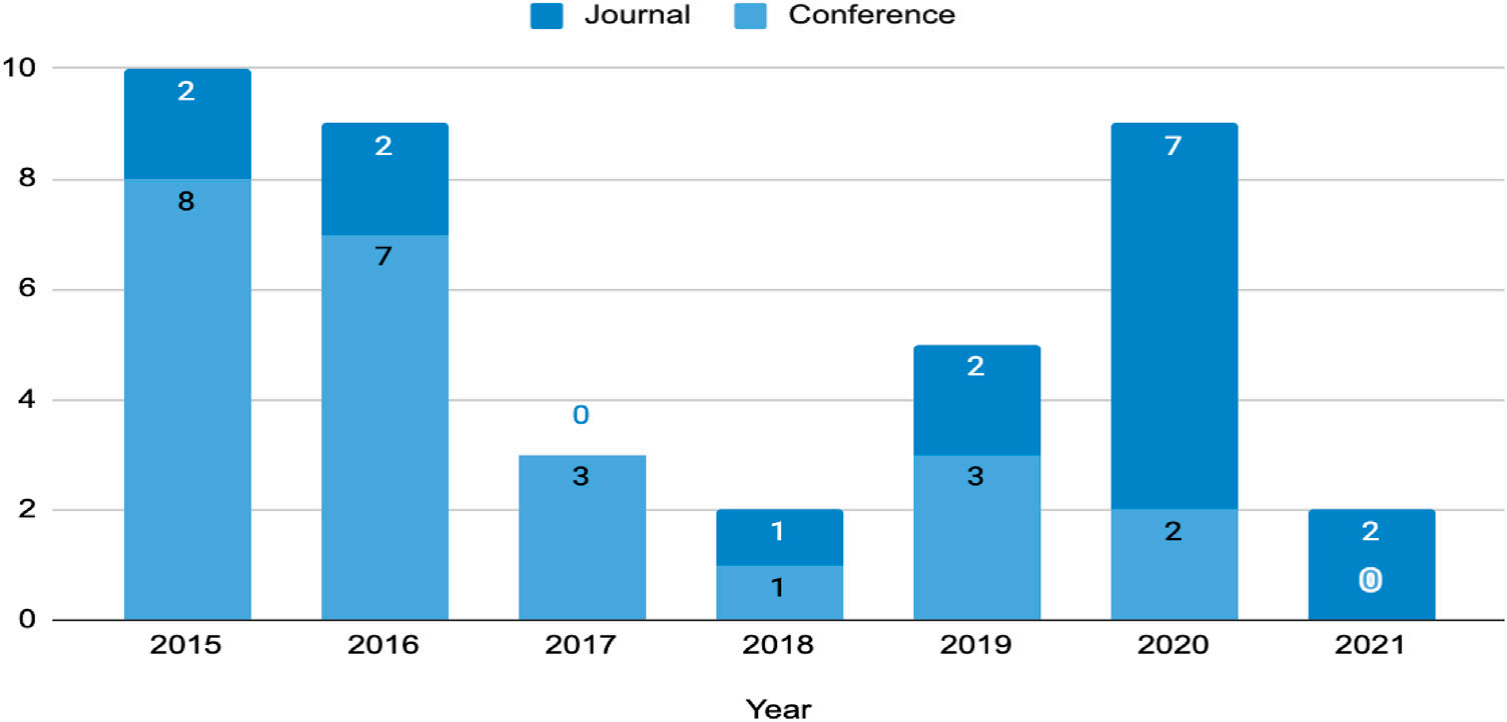
The number of collected conference and journal papers in 2015–2021.

We conduct quantitative analysis for answering the first research question, dealing with the techniques, tools, and architectures used to conduct sentiment analysis in MOOCs discussion forums. Figure 3 presents the relevant studies distributed according to year and database source. From the figure, it can be observed that the most relevant and selected studies is IEEE Xplore with 13 studies, followed by Scopus with 8 studies. Moreover, as can be seen from Figure 4, which illustrates the distribution of conference and journal papers, there has been an increasing trend of research works in journals in the last 2 years. During the previous years, most of the studies were published in conferences.

**FIGURE 5:**
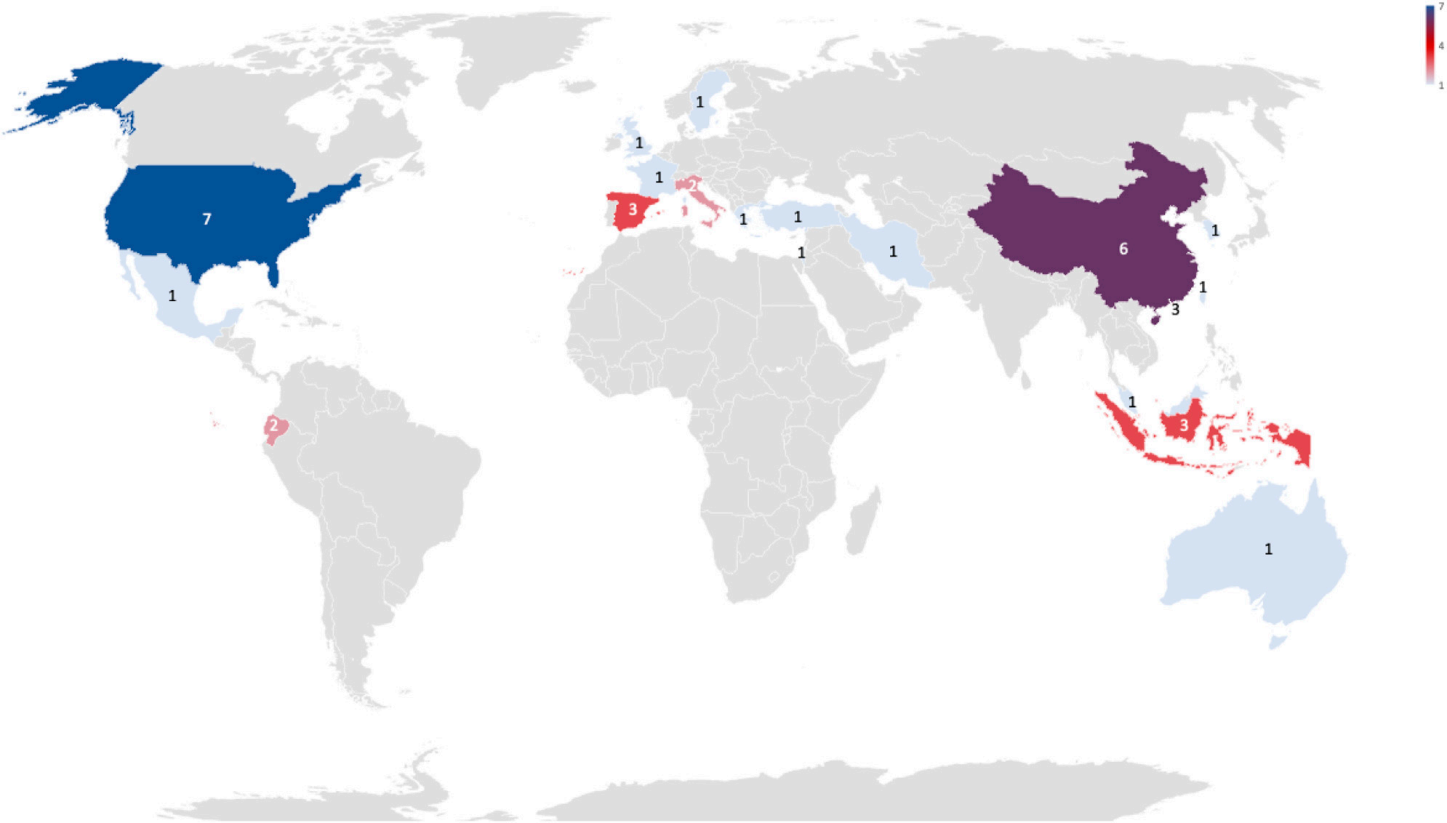
The number of collected papers across different regions/countries of first author.

By observing the country of origin of the first author, most of the works are from Asia with 17 papers, followed by Europe with 10 papers, and North America with 8 papers. In Asia, most of the studies are from China. Figure 5 shows the distribution by country.

When it comes to the techniques used to conduct sentiment analysis in MOOCs, they can be categorized mainly into four different groups, namely supervised, unsupervised, lexicon-based approach, and statistical analysis. Table 3 presents papers clustering based on learning approaches (techniques) that the authors have applied. In total, 21 papers used either supervised, unsupervised, and lexicon-based techniques or a combination among the three groups. Nine papers used statistical analysis while the rest of the papers did not explicitly specify the technique.

**TABLE 3:** Papers based on used technique/learning approach.

Technique (learning approach)	Related studies
Supervised	Lei et al. (2015), Shen and Kuo (2015), Lubis et al. (2016), Buenaño-Fernández et al. (2017), Estrada et al. (2020), Hew et al. (2020), Kastrati et al. (2020)
Unsupervised	Liu et al. (2017a), Yan et al. (2019)
Lexicon-based	Cobos et al. (2019b), Lundqvist et al. (2020)
Supervised and unsupervised	Moreno-Marcos et al. (2018)
Lexicon-based and supervised	Cobos et al. (2019a), Capuano et al. (2020), Capuano and Caballé. (2020), Dina et al. (2021), Onan. (2021), Qi and Liu. (2021), (Li et al., 2019)
Lexicon-based and unsupervised	Ezen-Can et al. (2015)
Lexicon-based and unsupervised or supervised	Badache and Boughanem (2014)
Statistical analysis	Dowell et al. (2015), Koutsodimou and Jimoyiannis. (2015), Au et al. (2016), Holstein and Cohen (2016), Lee et al. (2016), Liu. (2016), Liu et al. (2017a), Lee et al. (2020)
N/A	Sa’don et al. (2014), Crossley et al. (2015), O’Malley et al. (2015), Crossley et al. (2016), Nissenson and Coburn (2016), Rahimi and Khosravizadeh (2018), Martínez et al. (2019), Zhang et al. (2020)

In Table 4, the most frequently used supervised learning algorithms are shown. As can be seen, Neural Networks (NN) and Naïve Bayes (NB) were used most often in the reviewed studies, followed by Support Vector Machines (SVM) and Decision Tree (DT) algorithms.

**TABLE 4:** Most frequently used supervised learning algorithms.

Supervised learning algorithms	Related studies
Neural Networks (NN)	Estrada et al. (2020), Cobos et al. (2019a), Capuano et al. (2020), Capuano and Caballé (2020), Onan (2021), Qi and Liu (2021), Li et al. (2019)
Naïve Bayes (NB)	Badache and Boughanem (2014), Cobos et al. (2019a), Buenaño-Fernández et al. (2017), Moreno-Marcos et al. (2018), Onan (2021), Shen and Kuo. (2015)
Support Vector Machines (SVM)	Cobos et al. (2019b), Buenaño-Fernández et al. (2017), Dina et al. (2021), Moreno-Marcos et al. (2018)
Decision trees (DT)	(Hew et al. (2020)), Moreno-Marcos et al. (2018), Onan (2021)

Table 5, lists the use of lexicon-based approaches, which are also known as rule-based sentiment analysis. The most frequently used lexicons among the reviewed articles is VADER (Valence Aware Dictionary and Sentiment Reasoner), followed by TextBlob and SentiWordNet.

**TABLE 5:** Most frequently used lexicons.

Lexicon-based approach	Related studies
VADER	Cobos et al. (2019a), Cobos et al. (2019b), Lundqvist et al. (2020)
TextBlob	Cobos et al. (2019a), Cobos et al. (2019b)
SentiWordNet	Badache and Boughanem. (2014)

**FIGURE 6:**
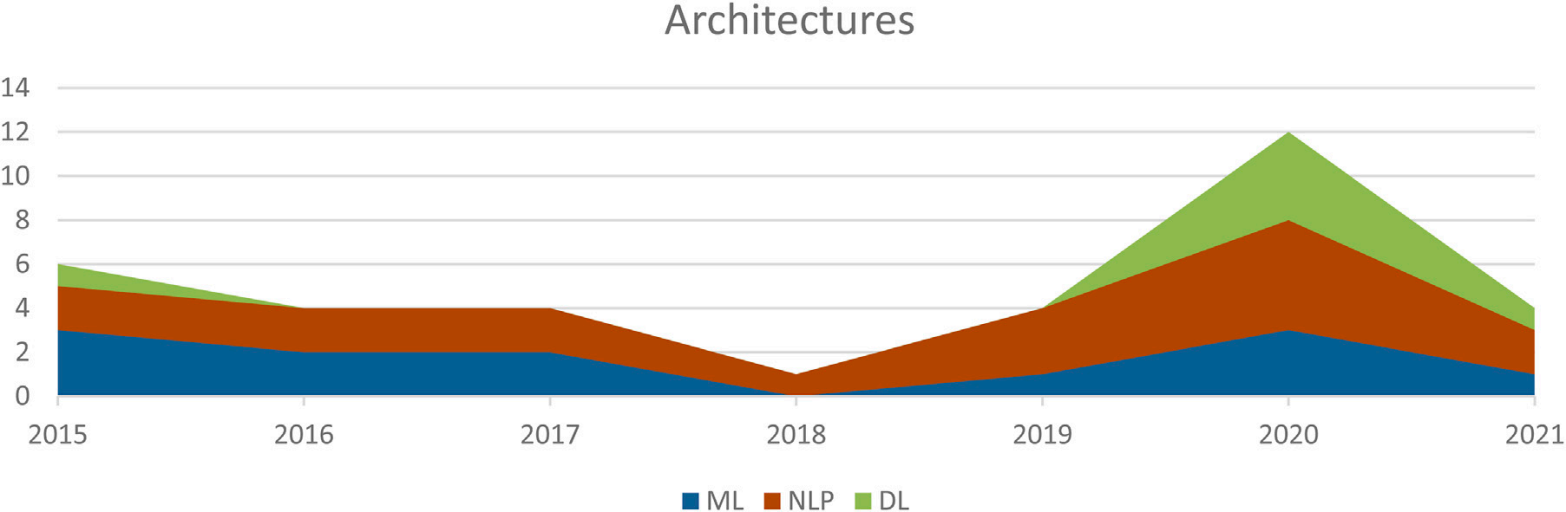
Distribution of architectures during 2015–2021.

Regarding the architecture, ML, DL and NLP were presented in the reviewed articles. Figure 6 illustrates that NLP and DL are most often used starting from 2020 onwards. Hence, NLP is used in seven papers, followed by DL with five papers.

**FIGURE 7:**
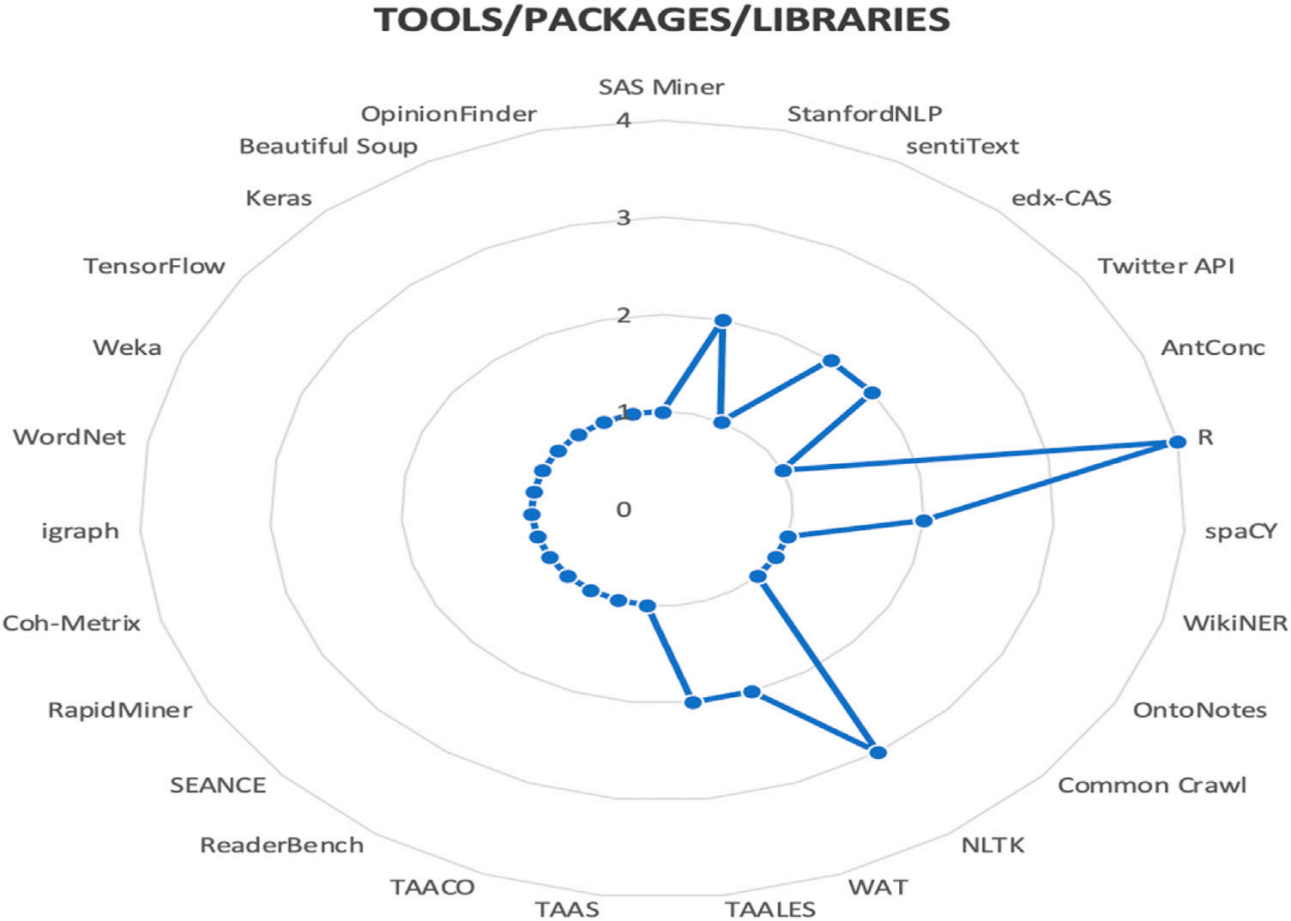
Tools/packages/libraries/used for sentiment analysis in the reviewed papers.

Figure 7 below shows the findings reviewed in the study with respect to the most frequently used packages, tools, libraries, etc. for the sentiment analysis task in MOOCs.

As presented in the figure, the most popular solution to conduct sentiment analysis is R and was used in four studies. Next, NLTK was the second most used platform. On the other hand, StanfordNLP, NLTK, spaCY, edX-CAS, WAT and TAALES represent the second category of most used solutions, each of them appearing in two different articles. The third group is composed of a variation of solutions which appear only once across the reviewed articles.

### Qualitative Analysis

To answer the second research question, the process continued with the strategy described by (Braun and Clarke, 2006). This encompasses an inductive thematic approach to identify common themes identified in every article. This process involves six phases: *familiarizing with data*, *generating initial codes*, *searching for themes*, *theme review*, *defining themes* and *naming themes*. Familiarization with the literature was reached during screening. The authors then inductively coded the manuscripts. The codes were collected in an Excel file to prepare for the upcoming steps. Further, the codes were grouped and consolidated in order to find and identify themes. Upon final agreement of themes and their definitions, a narrative through independent and collaborative writing and reviewing was built, following the recommendations from (Lincoln and Guba, 1985; Creswell and Miller, 2000). The overall process resulted in 6 themes, each discussed in detail in the discussion section. A summary of this assessment is presented in Table 6.

**TABLE 6:** Summary of identified themes.

Theme	Related papers
MOOC content evaluation	Lei et al. (2015), Au et al. (2016), Lee et al. (2016), Liu et al. (2017b), Li et al. (2019), Dina et al. (2021)
Review (feedback) contradiction analysis	Badache and Boughanem (2014), Liu et al. (2017a), Kastrati et al. (2020)
SA effectiveness	Ezen-Can et al. (2015), Koutsodimou and Jimoyiannis (2015), Holstein and Cohen (2016), Cobos et al. (2019b), Cobos et al. (2019a), Capuano et al. (2020), Capuano and Caballé (2020), Estrada et al. (2020), Hew et al. (2020), Onan (2021)
SA through social networks posts	Buenaño-Fernández et al. (2017), Dowell et al. (2015), Lee et al. (2020), Lundqvist et al. (2020), Moreno-Marcos et al. (2018)
Understanding course performance and dropouts	Crossley et al. (2015), Dowell et al. (2015), Crossley et al. (2016), Lubis et al. (2016), Nissenson and Coburn (2016)
MOOC design model evaluation	Lee et al. (2016), Liu (2016), O’Malley et al. (2015)

## Discussion

In this section, the types and trends of research conducted within each of the previously identified themes are explored and discussed. Finally, recommendations and suggestions for addressing the identified challenges are provided.

### MOOC Content Evaluation

In order to create relevant and useful insights for MOOC content development, course designers and learning analytics experts need to process and analyze a complex set of unstructured learner-generated data from MOOC discussion forums. The course content evaluations via sentiment analysis approaches can provide substantial indications to instructional designers and teachers to periodically evaluate the courses and introduce potential improvements.

In a study with a small sample of 28 students, the learners had a positive attitude and perception towards the quality of MOOC content (88.6%). Moreover, the text-mining based evaluation of the content conducted on the study also confirmed a high satisfaction on MOOC content. Here, the positive features included “interesting,” “easy,” and “duration of video is appropriate” (Au et al., 2016).

(Dina et al., 2021) explored the performance of a quantitative (SA based) model to measure the user preferences regarding the course content. The sentiment analysis classification has been done using Support Vector Machine. The accuracy, precision, recall, and F1 score were above 80%. Some of the positive features produced by this model were “course-good,” “course-interesting, ”“course-easy,” “course-understand,” “course-recommended,” and “material-good.” In another case study, a *learner decision journey* framework was proposed to analyze the MOOC content development, to understand the circular learning process, and to generate further insights for course improvements (Lei et al., 2015). The study showed the presence of posts with significant positive sentiment scores during the entire course, meaning that learners were positive towards the content and also in completing the course.

An application framework of an intelligent system for learner emotion recognition on MOOCs was proposed by (Liu et al., 2017a), where obtaining the learners’ emotion-topic feedback about content proved to be instrumental for teachers to analyze and improve their teaching pedagogy. Furthermore, an analysis of sentiments of MOOC learners’ posts via deep learning approach was conducted by (Li et al., 2019). The experiments in this study revealed that the approach could be effectively used to identify content related problems and to improve educational outcomes. In contrast to lexicon-based approaches, which were also evaluated in the study, deep learning models could further reduce the consumption of constructing sentiment dictionaries, among others.

### Review (Feedback) Contradiction Analysis

Although the learner-generated reviews and opinions have great practical relevance to educators and instructional designers, sometimes, learners’ comments tend to be contradictory (positive vs. negative), which creates difficulties for teachers to understand them. One possible explanation for such a contradiction is that MOOC learners are quite heterogeneous with different educational backgrounds, knowledge, and motivations (Nie and Luo, 2019). However, the large-scale comments, negative opinions and emotions in particular, can spread faster than positive ones (Pugh, 2001), and these could lead to dropouts. Only three studies were found to be focused on the contradiction analysis of MOOC reviews (Badache and Boughanem, 2014; Liu et al., 2017a; Kastrati et al., 2020).

An experimental study on the detection of contradictory reviews in Coursera based on the sentiment analysis around specific aspects was conducted by (Badache and Boughanem, 2014). Before extracting particular aspects according to the distribution of the emotional terms, the reviews were first grouped according to the session. Further, the polarity of each review segment holding an aspect was identified. The results of experiments with 2,244 courses and 73, 873 reviews revealed the effectiveness of the proposed approach towards isolating and quantifying contradiction intensity. Another aspect-based sentiment analysis framework tested and validated in Coursera dataset was proposed by (Kastrati et al., 2020). Researchers have achieved a high-performance score (F1 = 86.13%) for aspect category identification, which demonstrates the reliability and the comprehensiveness of the proposed framework.

Some other scholars also recommended a generative probabilistic model that extends Sentence-LDA (Latent Dirichlet Allocation) to explore negative opinions in terms of pairs of emotions and topics (Liu et al., 2017a). With this model, the detection precision of negative topics reached an acceptable accuracy rate of (85.71%). The negative comments were mainly revolving around learning content, online assignments and course certificates.

### SA Effectiveness

The effectiveness evaluation of sentiment analysis models was a key focus of much of the reviewed papers, especially those published after 2019. This could be due to the recent trends of making datasets available and the goals of the MOOC providers, because sentiment analysis techniques can shed more light towards improving enrollment and learning experience. During the period of 2015 and 2016, most of the works utilized the clustering models to group similar MOOC discussion forum posts, along with topic modeling to capture the topical themes (Ezen-Can et al., 2015). The main reason behind some works was also to increase satisfaction of teachers who themselves attend MOOCs to support their own professional development (Koutsodimou and Jimoyiannis, 2015; Holstein and Cohen, 2016).

However, most of the identified research papers that evaluated the effectiveness of the sentiment analysis models were published during 2019 and 2020 (Cobos et al., 2019a; Cobos et al., 2019b; Yan et al., 2019; Capuano and Caballé, 2020; Capuano et al., 2020; Estrada et al., 2020; Hew et al., 2020; Onan, 2021). (Cobos et al., 2019a; Cobos et al., 2019b) compared and measured the evaluation effectiveness of machine learning (SVM, NB, ANN) and NLP approaches (VADER, TextBlob) to extract features and perform text analysis. Their prototype was based on a content analyser system for edX MOOCs. Another group of researchers conducted a relevant study by applying unsupervised natural language processing techniques to explore students’ engagement in Coursera MOOCs (Yan et al., 2019). Further, they evaluated the performance of LDA, LSA (Latent Semantic Analysis) and topic modelling to discover the emerging topics in discussion forums and to investigate the sentiments associated with the discussions.

After 2019, along with the machine learning and natural language processing techniques (Hew et al., 2020), researchers started to use and measure the effectiveness of deep learning architectures for sentiment analysis on MOOCs that exhibit an improved performance compared to conventional supervised learning methods (Capuano and Caballé, 2020; Capuano et al., 2020; Estrada et al., 2020; Onan, 2021). The most widely used deep learning approaches by researchers are CNN (Convolutional Neural Networks), LSTM (Long Short-Term Memory), BERT (Bidirectional Encoder Representations from Transformers), and RNN (Recurrent Neural Networks).

### SA Through Social Networks Posts

The research has demonstrated that social networking sites can significantly impact the interaction of learners with courses (Georgios Paltoglou, 2012). With the growing popularity of social networking, sentiment analysis has been used with social networks and microblogging sites, especially Twitter or blogs (Hong and Skiena, 2010; Miller et al., 2011). However, the nature and the structure of the texts published in social networks is largely scattered and unstructured. Therefore, many researchers have adopted various social media mining approaches to investigate the sentiments of Twitter messages related to MOOC learning (Shen and Kuo, 2015; Buenaño-Fernández et al., 2017). The main goal of these studies was to explore the students’ tweets (positive and negative) about the course, and to evaluate instructors and the educational tools used in the course. (Lundqvist et al., 2020) employed sentiment analysis to investigate the online comments of MOOCs where VADER (Valence Aware Dictionary for sEntiment Reasoning) sentiment algorithm was used. Sentiment ratings from 90,000 social media based posts are included in VADER. From all analyzed comments, it was revealed that there exists a correlation between sentiments of the posts and the feedback provided about the MOOC. Moreover, 78% of students were positive towards the MOOC structure. Almost all identified papers were using Twitter to explain the insights of MOOCs from social media platforms. Future invastigations may also consider other platforms, such as Facebook or Youtube and compare with findings obtained for Twitter.

### Understanding Course Performance and Dropouts

The major challenge of MOOCs is the massive dropout or retention (Chen et al., 2020). In parallel with the factors, like demographic characteristics, interaction, self-reported motivation, and commitment attitudes, this paper stresses that learners’ lack of self-regulation might create cliffhangers that should be instantly conquered to benefit from the MOOCs.

The best way to predict the prospective dropouts is to analyse the reactions within SA and to extract those keywords that reveal that the dropouts are predominantly related with the course performance. Such analysis was performed in more detail in five of the eligible papers (Crossley et al., 2015; Dowell et al., 2015; Crossley et al., 2016; Lubis et al., 2016; Nissenson and Coburn, 2016), showing that many researchers have been intrigued by the poorer course performance and decreased interest to persist in the course. Three of them concentrate on the discussion forums (Crossley et al., 2015; Dowell et al., 2015; Crossley et al., 2016). While (Crossley et al., 2015) embraces the language used in the discussion forums as a predictive feature of successful class completion, (Crossley et al., 2016), also examines the online clickstream data and the language. The last one (Dowell et al., 2015) additionally examines the social position of learners as they interact in a MOOC. Last two papers that investigate the language to understand learner’s performance and dropouts are mainly focused on the attributes that contribute towards predicting the successful course completion (Lubis et al., 2016), (Nissenson and Coburn, 2016). They both extract the attributes that exhibit learners’ satisfaction only, rather than those factors that might suppress learners from continuing their studies in the MOOCs. (Lubis et al., 2016) is even more optimistic, and never explicitly mentions dropouts. This is extremely good news, knowing that the analysis was done over 20,000 reviews crawled from class central websites containing 1900 topics.

The general objective of this cluster of papers is to analyse the sentiment analysis by examining the language used in it. Depending on the research hypotheses in them, the attributes used to explore learners’ opinion vary from moderately pessimistic to very optimistic. Undoubtedly, several more papers implementing the same approach will contribute to increasing the impact of MOOCs on education and minimizing the risk of premature retention.

### MOOC Design Model Evaluation

As elaborated in the *MOOC content evaluation*, the evaluation of MOOC content is crucial for the evolution of the MOOCs, since it determines and proposes the necessary improvements that are inevitable to extend MOOCs lifecycle. Quite unexpectedly, several of the surveyed papers suggested an improvement of the design model, as a complementary element that is essential to keep the MOOC active and prosperous. In the first place, they notice that there are many differences of the language used for MOOC supported online and real classes (Rahimi and Khosravizadeh, 2017). The distinction is done including both, the text and the speech analysis. More profoundly, the research in (Qi and Liu, 2021) proposes LDA for mining of the student generated reviews with an ultimate aim to objectively and accurately evaluate the indicators providing reliable references for both, the students and the educators. Based on the established means for text mining of sentiment analysis and the profound processing of the results, reorganization of the model can start. The strategy is proposed in (Lee et al., 2016). By introducing 11 design criteria for organization of the model, this paper examines the MOOC characteristics and their impact on satisfaction of instructor and learner.

The last two papers from this cluster are topic specific. (Liu, 2016) explores a new model based on English for Specific Purposes for the course of metallurgical English. To strengthen the approach, authors suggest a symbiosis between MOOCs and flipped classrooms, in the light of the course purpose, content, teaching organization and finally, teachers’ evaluation. By making the synergy between both teaching methodologies, they believe that the course will significantly advance. (O’Malley et al., 2015) goes one step forward, it suggests a reconstruction of MOOCs into a virtual laboratory using video and simulations. This is an outgoing project, intended to adapt online delivery format for a campus-based first year module on Physical chemistry at University of Manchester. The experience of merging MOOC with a virtual laboratory proved its efficiency. Improvement of the content needs an improvement of the design model.

On many occasions, the improvement of a product means an improvement of the technology that enables it. The last theme of this survey proves this claim. It can be done by adding new features, such as the flipped classroom (Liu, 2016) and the virtual laboratory (O’Malley et al., 2015). This extension should be done steadily and carefully to avoid the risks of ruining the product. To enable the extensions, it is inevitable to maintain the existing features. They can be assessed by implementing the design criteria (Lee et al., 2016). However, all the improvements must be appreciated by their end users, the learners and the teachers. The evaluation includes SA performed using the techniques proposed in (Qi and Liu, 2021; Rahimi and Khosravizadeh, 2017). The last, but not the least is to support the philosophy of continuous improvement. This returns the sentiment analysis to the first theme: MOOC content evaluation, and then continues with all the remaining themes, creating a never-ending lifecycle for evaluation of MOOCs.

### Recommendations and Future Research Avenues

When considering the MOOC content evaluation of the relevant studies documented in our reviewed sample, overall, there is a favorable rating of course content among learners. As can be seen from the above discussion, most research on MOOC content evaluation is focused on the learner feedback, however, future scholars could also consider investigating the teacher’s feedback/perspective towards the content development, teaching pedagogy, experience, and assessment, among others. Moreover, it would be also interesting to consider exploring the results provided by sentiment analysis techniques in collaboration with the instructors of the MOOC course to know if their proposed materials could be improved.

Throughout the reviewed papers, imbalanced datasets with underrepresented categories were evidenced. Therefore, a recommendation for researchers to achieve performance improvement is by applying data augmentation techniques. Classifier performance can be improved by adopting more advanced word representation approaches like contextualized embeddings as well as classical NLU (Natural Language Understanding) techniques, such as part-of-speech, parsing, etc.

Furthermore, exploring the relationship between polarity markers and other feeling labels or emotions could be beneficial towards better identification and addressing of the issues related towards the target subject, as has been studied in many relevant text-based emotion detection works (Acheampong et al., 2020).

A considerable number of reviewed papers failed to report on how the results were standardized in terms of participant numbers and characteristics, course subject and context, accuracy, and metrics of SA approaches. Hence, we consider that a special focus should be placed towards enhancing the transparency of the research results. This could be beneficial and advantageous to other researchers when conducting comparative performance analysis between various sentiment analysis approaches.

Some of the studies related to recognition of polarities and emotions in MOOCs are conducted in laboratory settings and utilize a limited set of algorithmic solutions and techniques. However, more standardized investigations are needed to be conducted with students using more algorithms with different configurations of hyper-parameters and layers. This way, standardization will contribute to assuring the quality, safety, and reliability of the solutions and techniques designed for sentiment analysis in MOOC learning environments. In addition, there is also a lack of standardized datasets available for the evaluation of sentiment analysis models in MOOCs. Most of the researchers have used publicly available datasets of Coursera, edX, FutureLearn, and even datasets from their own institutions (Ezen-Can et al., 2015; Moreno-Marcos et al., 2018; Cobos et al., 2019a; Yan et al., 2019; Estrada et al., 2020; Lee et al., 2020). The absence of standardized datasets plays a negative role when benchmarking or comparing algorithmic solutions of different researchers. It is also worth mentioning that researchers used datasets from predominantly computer science courses to evaluate and explore sentiment analysis of students’ feedback in MOOCs (Moreno-Marcos et al., 2018; Estrada et al., 2020; Lee et al., 2020; Lundqvist et al., 2020). Thus, the research is mainly limited to one academic field.

It was also observed that the reviewed research papers have not taken into consideration different types of MOOCs, such as cMOOCs, xMOOCs, or sMOOCs. In the future, sentiment analysis of students’ feedback should also consider different types of MOOCs.

In addition, if enough suitable (standardized) datasets could have been available, it can be interesting to introduce more meticulous RQs and to try a meta-analysis, or even an advanced systematic quantitative literature review, involving more complex statistical operations. This could, however, serve as an insightful idea for a future work.

## Conclusion

Although introduced almost 75 years ago, sentiment analysis has recently become a very popular tactic for gathering and mining the subjective information from end users of various services. Implementing popular NLP, statistical and ML techniques, sentiment analysis grows into a cost-effective tool to distil the sentiment patterns that reveal the potential challenges of the existing services, and at the same time, identify new opportunities and improvements. Its extensive implementation contributed to increased accuracy and efficiency wherever it was used.

The use of sentiment analysis techniques to understand students’ feedback in MOOCs represents an important factor to improve the learning experience. Moreover, sentiment analysis can be also applied to improve teaching by analyzing the learners’ behavior towards courses, platforms, and instructors.

To evaluate these claims, a PRISMA directed systematic review of the most recent and more influential scholar publications has been done. The review has gone through an exhaustive quantitative and qualitative stepwise filtering of the initial corpus existing of 440 articles that fulfilled the search criteria associated with PICO(C). Together with the briefly introduces methodology, search strategy and data selection, the authors have also tackled the potential limitations of the proposed approach. After these introductory sections, the paper thoroughly presents the quantitative results for 40 relevant papers, starting from the process of analysing relevant contributions, their contribution in academic databases and annual and geographical distribution, then makes an overview of the implemented sentiment analysis technique and supervised learning algorithms and lexicons, to end up with the distribution of architectures, tools/packages/libraries/used for sentiment analysis in the reviewed papers. It is worth mentioning that from 2019 onwards researchers have started to apply deep learning in combination with NLP approaches to analyze the sentiments of students’ comments in MOOCs.

Qualitative analysis identified the following six major themes being used in the reviewed papers: MOOC content evaluation, review (feedback) contradiction analysis, SA effectiveness, SA through social networks posts, understanding course performance and dropouts, and MOOC design model evaluation. As part of this analysis, each theme was carefully presented and illustrated with the corresponding filtered references that fulfil all the criteria.

We believe that this work could be a good inspiration for future research, and that will provide readers with interesting information in a wide context about the current trends, challenges, and future directions in the field.

## Data Availability

The original contributions presented in the study are included in the article/supplementary material, further inquiries can be directed to the corresponding author.
